# Purple Urine Bag Syndrome: An Unusual Presentation of Urinary Tract Infection

**DOI:** 10.7759/cureus.16319

**Published:** 2021-07-11

**Authors:** Ravi Kumar, Kanchan Devi, Deepak Kataria, Jai Kumar, Ishtiaq Ahmad

**Affiliations:** 1 Internal Medicine, Jinnah Sindh Medical University, Karachi, PAK; 2 Internal Medicine, Shaheed Mohtarma Benazir Bhutto Medical University, Larkana, PAK; 3 Internal Medicine, Liaquat University of Medical and Health Sciences, Jamshoro, PAK; 4 Internal Medicine, Khyber Medical College, Peshawar, PAK

**Keywords:** purple urine bag, purple urine bag syndrome, tryptophan, urinary catheters, indigo, indirubin

## Abstract

Purple urine bag syndrome (PUBS) is a very rare phenomenon strongly associated with long-term indwelling catheterization that results in an increased risk of urinary tract infection. The color change in the urine bag results from the altered metabolism of tryptophan into color pigments by certain bacteria which produce sulfates and phosphates enzymes. Although it is benign in nature, PUBS results in greater anxiety among patients and their families. The most important risk factors include long-term catheterization, female gender, chronic constipation, old age and bed-bound patients. Here, we present a case of PUBS in a middle-aged woman with a history of the neurogenic bladder that needed long-term catheterization along with chronic constipation.

## Introduction

Purple urine bag syndrome (PUBS) is a rare clinical presentation of urinary tract infection, which results in purple discoloration of urine bag and tube. It mostly indicates ongoing urinary tract infection, where certain bacteria produce enzymes that metabolize tryptophan into indigo (blue) and indirubin (red) pigments to produce a purple color of urine. PUBS is mostly seen in elderly female patients with an indwelling catheter and chronic constipation. It was first reported by Barlow and Dickson in 1978 [1]. Although it is benign in nature and only changes occur in the color of urine from yellow to purple, PUBS results in greater anxiety in patients. Here, we report this case, which was seen in middle age elderly female patient with long-term catheterization and chronic constipation presented to the outpatient clinic at The Kidney Center Karachi, Pakistan.

## Case presentation

**Figure 1:**
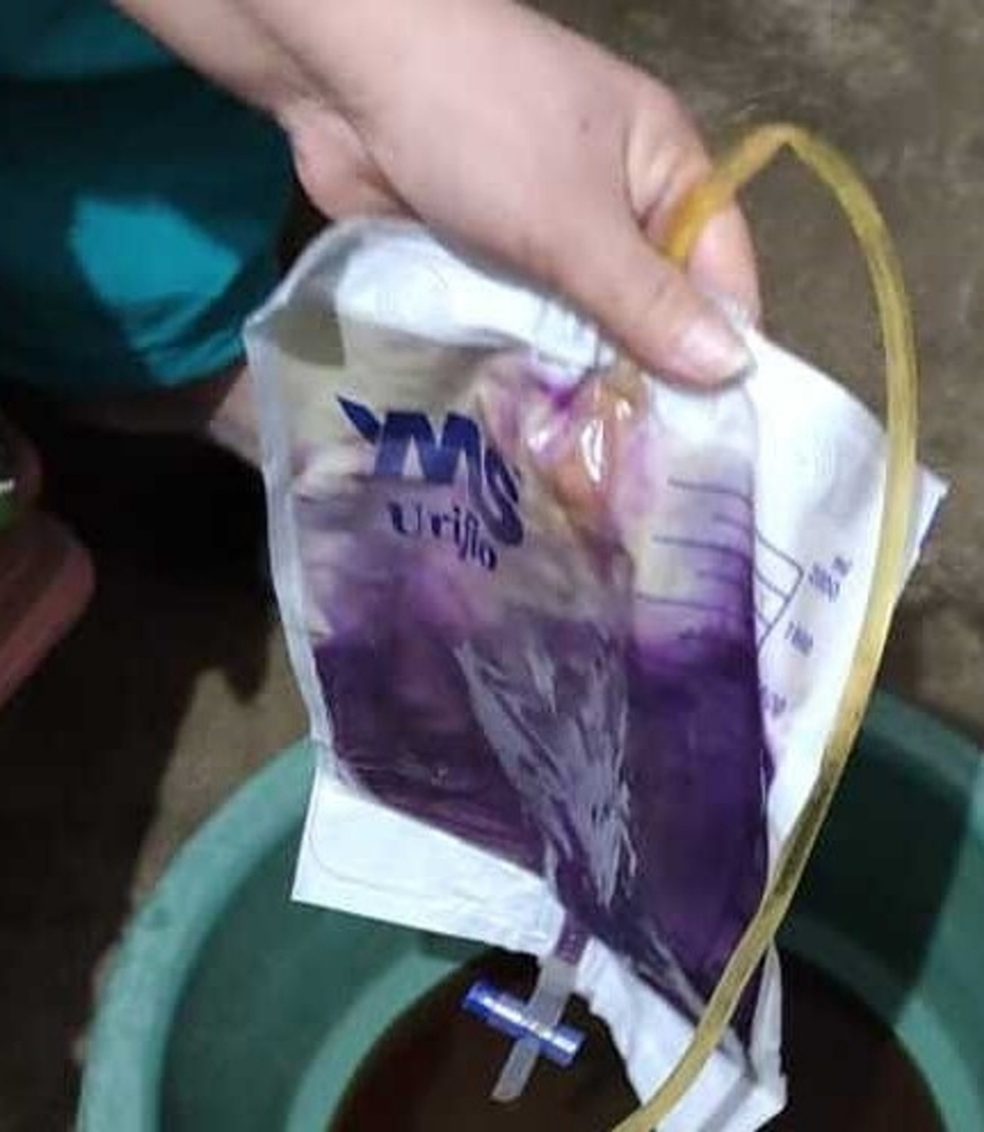
Purple Urine Bag Syndrome

A 52-year-old female with a known case of neurogenic bladder and chronic constipation presented to the outpatient nephrology clinic with complaints of nausea, fever and purple discoloration of the urine bag. The patient was known to the nephrology and urology team with a history of trauma four years back that resulted in the neurogenic bladder. Since then, she was on indwelling catheterization and visits the emergency department every month for a change of Foley catheterization. She reported that she had an on and off 100 F fever for the last three days, which was treated at home by taking acetaminophen 1g as needed. She also reported purple discoloration of urine in the bag (Figure 1) that appeared suddenly. The patient does not have burning micturition, supra-pubic pain, vomiting and chills. Her physical examination was normal and labs revealed increased white cell counts. Her Foley catheterization was changed along with a new bag and a fresh urine sample was sent for urine analysis along with urine culture. The patient was empirically treated with antibiotic cefixime (third-generation cephalosporin) 400mg once a daily for five days. Patient-reported resolution of purple discoloration within three days of antibiotic therapy and was continued on this therapy for a total of 10 days. Her urine analysis was positive for nitrates, leucocyte esterase with alkaline urine. Her urine culture showed growth of Escherichia coli (>100,000/mL after 48 hours), which was sensitive to cefixime, ciprofloxacin, amikacin, gentamycin, nitrofurantoin and colistin. The patient was seen on the 10th day of her follow-up after completing her antibiotic therapy, which resulted in the complete resolution of her symptoms. 

## Discussion

Many females in their lifetime can have a urinary tract infection and presentation can vary from mild symptoms to severe urosepsis. PUBS is a very rare phenomenon and unusual presentation of urinary tract infection that is mostly seen in elderly female patients with long-term indwelling catheterization, chronic constipation especially in those patients who have very limited mobility. Other risk factors include old age, female gender, alkaline urine and the use of plastic bags [1,2]. In our patient, indwelling catheter and chronic constipation were present. However, patients with risk factors for developing PUBS have more frequent episodes of urinary tract infection even than this condition appears to be very rare in clinical presentation.

The exact mechanism of PUBS is related to the conversion of tryptophan into color pigments indigo and indirubin, which gives purple color to urine [3,4]. Normally intestine bacteria convert tryptophan into indoles, which go into the intrahepatic circulation where it is converted into indoxyl sulfate that is excreted in the urine. But if the patient had a urinary tract infection in the presence of alkaline urine, certain bacteria produce indoxyl sulfatase enzyme that converts indoxyl sulfate into indoxyl, which changes to indirubin (red) and indigo (blue), and the mixture of these two colors produces purple discoloration [3-5]. Chronic constipation also alters gut motility that resulted in an increased number of bacteria that digest more tryptophan and more pigments are released into urine resulting in the purple color of urine. The bacterial species producing these enzymes that are most commonly associated with PUBS are E. coli, Providencia stuartii, Providencia rettgeri, Klebsiella pneumonia, Proteus sp., Enterococcus sp., Morganella morganii, Pseudomonas aeruginosa, and certain gram-negative bacteria [4-6].

The management of PUBS mostly relies on identifying the underlying cause, which in most cases is bacterial urinary tract infection and constipation. Changing the Foley catheter with a new bag and treating the infection with antibiotics resolve the symptoms within one week. The most common antibiotic used for PUBS is ciprofloxacin (a quinolone), which is considered appropriate empirical therapy [7]. However, in our case, cefixime worked very well. Using non-plastic urine bags is also considered a prevention method for PUBS [3]. Many experts believe that increasing mobility and implementing safe and hygienic methods during catheterization will reduce the chances of developing purple urine bag syndrome. Treating other risk factors like constipation will decrease the chances of developing this complication.

## Conclusions

PUBS is a rare presentation of underlying urinary tract infection that is mostly seen in chronically catheterized, bedridden, constipated elderly patients and the sudden appearance of purple color in urine can be anxiety-provoking to patients and their families. The aim of writing this case report is to make aware primary care physicians in Karachi, Pakistan, to recognize this condition and treat urinary tract infections with appropriate antibiotics, along with using non-plastic bag results in complete resolution of the color of urine.
